# Using Multiple Imputations to Accommodate Time-Outs in Online Interventions

**DOI:** 10.2196/jmir.2781

**Published:** 2013-11-21

**Authors:** Susan M Shortreed, Andy Bogart, Jennifer B McClure

**Affiliations:** ^1^Group Heatlh Research InstituteBiostatistics UnitSeattle, WAUnited States; ^2^University of WashingtonDepartment of BiostatisticsSeattle, WAUnited States; ^3^Group Health Research IntituteSeattle, WAUnited States

**Keywords:** online interventions, engagement, time spent online, multiple imputations, automatic time-out, smoking cessation, utilization, behavioral research, Internet

## Abstract

**Background:**

Accurately estimating the period of time that individuals are exposed to online intervention content is important for understanding program engagement. This can be calculated from time-stamped data reflecting navigation to and from individual webpages. Prolonged periods of inactivity are commonly handled with a time-out feature and assigned a prespecified exposure duration. Unfortunately, this practice can lead to biased results describing program exposure.

**Objective:**

The aim of the study was to describe how multiple imputations can be used to better account for the time spent viewing webpages that result in a prolonged period of inactivity or a time-out.

**Methods:**

To illustrate this method, we present data on time-outs collected from the Q^2^ randomized smoking cessation trial. For this analysis, we evaluate the effects on intervention exposure of receiving content written in a prescriptive versus motivational tone. Using multiple imputations, we created five complete datasets in which the time spent viewing webpages that resulted in a time-out were replaced with values estimated with imputation models. We calculated standard errors using Rubin’s formulas to account for the variability due to the imputations. We also illustrate how current methods of accounting for time-outs (excluding timed-out page views or assigning an arbitrary viewing time) can influence conclusions about participant engagement.

**Results:**

A total of 63.00% (1175/1865) of participants accessed the online intervention in the Q^2^ trial. Of the 6592 unique page views, 683 (10.36%, 683/6592) resulted in a time-out. The median time spent viewing webpages that did not result in a time-out was 1.07 minutes. Assuming participants did not spend any time viewing a webpage that resulted in a time-out, no difference between the two message tones was observed (ratio of mean time online: 0.87, 95% CI 0.75-1.02). Assigning 30 minutes of viewing time to all page views that resulted in a time-out concludes that participants who received content in a motivational tone spent less time viewing content (ratio of mean time online: 0.86, 95% CI 0.77-0.98) than those participants who received content in a prescriptive tone. Using multiple imputations to account for time-outs concludes that there is no difference in participant engagement between the two message tones (ratio of mean time online: 0.87; 95% CI 0.75-1.01).

**Conclusions:**

The analytic technique chosen can significantly affect conclusions about online intervention engagement. We propose a standardized methodology in which time spent viewing webpages that result in a time-out is treated as missing information and corrected with multiple imputations.

**Trial Registration:**

Clinicaltrials.gov NCT00992264; http://clinicaltrials.gov/ct2/show/NCT00992264 (Archived by WebCite at http://www.webcitation.org/6Kw5m8EkP).

## Introduction

### Tracking Exposure Time to Content

As Internet-based behavioral interventions become more prevalent, it is increasingly important that researchers understand how people interact with these programs, including the time participants spend viewing individual content pages and interacting with the program overall [1-3]. Exposure time is one of several important proxies of engagement and could be an important mediator of the programs’ intended effects on participants’ knowledge, attitudes, and behavior.

Exposure time can be tracked by monitoring when each webpage is opened or exited or when the browser itself is closed. More sophisticated software can further assess activity on a particular webpage by tracking keystrokes or mouse clicks, but no software is able to distinguish when a user is actively reading or viewing a page versus engaged in other activities in their surroundings. Moreover, there are limitations on tracking activities such as viewing content in separate browsers or windows or even working concurrently in other open programs or applications. In all cases, the result will appear to be long periods of inactivity on the program webpage.

A common strategy for dealing with these extended periods of inactivity has been to time out the program after a prespecified time (eg, 30 minutes) [4-11]. This strategy makes sense as a means for closing out the program, but it would be misleading to rely on the time-stamped data from these timed-out periods as an indicator of how long participants were actually exposed to the program content in the open webpage. Other researchers have allowed long page views with no time-out feature, but then truncate the assumed actual viewing time after the fact for analytic purposes [5-7,12,13]. As these two approaches are equivalent for the purpose of measuring time spent online, we treat them identically and refer to each as a “time-out.”

Unfortunately, neither of the approaches above is ideal when trying to estimate the time participants were actively viewing online content. Each will likely either over- or underestimate the true viewing time. The actual length of time an individual spent engaged with the webpage is unknown, resulting in missing information. Consequently, excluding all page views that result in a time-out is the same as a complete case analysis and assigning an arbitrary length of time is the same as a single, uninformed imputation method. It is well-known that complete case analyses can result in bias and a reduction in power, as can single imputation [14-16]. As an alternative analytic approach, we recommend using standard missing data methods, in particular multiple imputations (MI), to accommodate long periods of inactivity or time-outs when analyzing time spent online.

Multiple imputations is a flexible and straightforward approach to accommodating missing data, which uses available observed information to predict values for missing information. Standard software exists and simple formulas can be used to incorporate multiple imputations into an analysis. We outline how to implement multiple imputations methods, reviewing standard formulae, to accommodate page views that resulted in a time-out. As an example, we use data collected from a randomized trial of an online smoking cessation intervention called the “Questions about Quitting*”* (Q^2^) trial [10,17]. Using data from this trial, we demonstrate how the method chosen for dealing with time-out data can significantly affect conclusions drawn about program exposure.

### The Questions About Quitting Trial

The Q^2^ trial was a collaboration between the Group Health Research Institute in Seattle, Washington, and the University of Michigan Center for Health Communications Research in Ann Arbor, Michigan. Detailed information about the study design and methods have been published elsewhere [17]. In brief, adult smokers were recruited from a large regional health plan population and invited to participate in a randomized clinical smoking cessation trial; however, participants did not have to have an interest in quitting smoking to enroll. The primary aim of this full factorial randomized trial [18] was to assess the effects of contrasting levels of four specific design features or factors, on smokers’ abstinence and utilization of adjunct treatment (counseling and pharmacotherapy) available to them through their health insurance. The effects of the contrasting levels of each design factor on program engagement were also explored and have been published [10].

Participants in this trial were randomized to one of 16 different combinations of the levels of the four design factors, with half of the participants assigned to one of two contrasting levels of each factor. Randomization was stratified by a baseline measure of a participant’s readiness to quit smoking. The four factors and the two contrasting levels of each were message tone (prescriptive vs motivational); navigation autonomy (dictated vs not dictated); proactive email reminders (yes vs no); and availability of testimonials (yes vs no). Here, we focus on comparing the impact of the two contrasting levels of message tone on program engagement, as measured by total time spent viewing online intervention content assessed during the first two months after study enrollment. Half of the participants were randomized to receive intervention content written in a prescriptive message tone, and half were randomized to an intervention written in a motivational tone. Intervention content written in a prescriptive tone was didactic and directly advised smokers to quit smoking and specified how to achieve this goal. In contrast, motivational messaging was written in a tone consistent with the main principles of motivational interviewing (express empathy, develop discrepancy, roll with resistance, support autonomy, and self-efficacy) [19].

The Q^2^ program collected automated tracking data each time participants visited the intervention website. This automated collection process recorded the date and time each participant visited the website and individual date/time stamps every time a content page was accessed or left by logging out of the intervention website, closing the browser, or moving to a different intervention webpage or an external webpage in the same browser window. The Q^2^ online intervention included an automatic time-out feature that logged participants out of the program after 30 minutes of inactivity.

## Methods

### Multiply Imputing Page View Times

Missing information is often classified according to the assumed missing data generating process, that is, the determinants that affect the probability that a particular data element is missing or observed. There are three general missing data generating processes: missing completely at random (MCAR), missing at random (MAR), or not missing at random (NMAR) [15,20]. MCAR assumes the probability that a data element is missing is independent of both observed and unmeasured information. This is unlikely to occur in practice and is the only situation in which a complete case analysis is unbiased (a reduction in power always occurs). The less restrictive MAR generating process assumes that the probability of a data element being missing depends on observed information, while NMAR means that the probability of missingness is dependent on both observed and unmeasured information.

Multiple imputations is a flexible and straightforward approach for accommodating missing data. Imputation methods estimate predictive models using observed information and replace missing data elements with samples from the estimated predictive models. Multiple imputations methods are preferred over single imputation [15,16,21] and repeatedly utilize estimated predictive models to create several complete datasets. Each complete dataset is then analyzed as if all information was observed and information is combined across each of the completed datasets.

There are two common approaches to estimate predictive models when multivariate imputation models are needed (ie, when more than one variable contains missing data or one longitudinal variable has missing information over time). One approach assumes a joint predictive distribution over all recorded variables [22,23], and the other method estimates separate conditional predictive models for each variable with missing information separately [24-26]. The second method is called multiple imputations by chained equations (MICE) or fully conditional specification and is growing in popularity due to its computational efficiency and flexibility. The MICE procedure can easily accommodate binary, categorical, and continuous variables as well as more complex data challenges such as bounded variable values and imputing information for subsets of individuals. For these reasons, we use MICE to impute missing page view times (ie, times for page views that timed out).

MICE methods cycle through each variable with missing information estimating regression models for each variable. Missing values are then replaced with samples from these regression-based predictive distributions, which include the appropriate random error. There are several built-in and stand-alone software packages that implement the MICE procedure [27-30]. MICE algorithms begin by imputing all missing information with naive values (eg, median of observed values of variable); then, the first variable (variable 1) containing missing information is considered (usually the variable with the least amount of missing data). A regression-based predictive model is estimated using observed values of variable 1 and observed and naively imputed values of all other variables selected as predictors. Usually all other variables are used as predictors, unless the analyst chooses to restrict the set of predictors [31,32]. The naively imputed values from variable 1 are replaced with imputations drawn from this predictive model, and the procedure continues on to the second variable with missing information (variable 2). A predictive model is estimated using the observed values of variable 2, the observed and newly imputed values of variable 1, and the observed and naively imputed values of all other predictors. The naively imputed values of variable 2 are then replaced with imputations drawn from this newly estimated imputation model. The imputation process cycles through all the variables that contain missing information replacing the naively imputed missing values with draws from newly estimated imputation models. When the MICE algorithm has cycled through all of the variables with missing information, this is called one “iteration”. The cycle is then repeated, replacing the imputed values from the first iteration with imputations from newly estimated predictive models in the second iteration. Several iterations of MICE are used to ensure that the imputations have “stabilized”, such that the order in which the variables were cycled through no longer affects the imputation values [24,31].

The iterative nature of the MICE algorithm provides both strengths and weaknesses. While the MICE procedure has proven useful in practice, it does not have the solid theoretical justification of alternative imputation methods. For example, convergence (ie, imputation values that “stabilize”) is not guaranteed [24,25]. It is also possible that conditional imputation models will be estimated such that there exists no joint multivariate distribution that is consistent with all conditional distributions. While these drawbacks may give rise to valid theoretical concerns, it appears that they are generally not a concern in practice [21,24,33,34], and MICE is increasingly being used to accommodate missing data in analyses [31,35-37].

Once *M* completed datasets have been created, each completed dataset is used to calculate the estimate of interest (see #1 in Multimedia Appendix 1), where the subscript *m* is used to denote that the estimate corresponds to the *m*-th completed dataset. The average of the *M* estimates (see #2 in Multimedia Appendix 1) is used as the estimate for the parameter of interest. Rubin developed a straightforward formula for estimating the standard errors of the multiple imputations estimators that accounts for the traditional sampling variability of the estimator and the added variability due to the imputation process [15,38,39]. Rubin’s formula can be used to calculate the standard error for most standard estimators. It is a function of the M complete data standard errors (*W*_*1,..M*_) and the variability between the complete data estimates across the M imputations (*B*_*M*_). Let *W*_*M*_ be the standard error of the complete data estimator in the *m*-th imputed dataset, then Rubin’s formula for the standard error of the imputation estimator appears as in #3 in Multimedia Appendix 1 [15,38]. In practice, analysts usually use 5-10 imputations as this has been shown to be sufficient to correctly capture the variability in the imputation estimator [39].

We generated five complete datasets with all missing page view times replaced with samples from estimated conditional imputation models. Imputation models were assumed to be normal distributions after log transforming the page view times with means and appropriate standard deviations estimated from linear regression models. We structured the data in a wide format with each person representing one row in the dataset and multiple webpage views represented by multiple columns. A new imputation model was estimated for each repeated page view. We used observed page view times for estimating imputation models and only imputed times for those page views that were observed but that resulted in an automatic time-out. Linear regression models were used to specify the mean of each of the conditional predictive distributions with the following predictors: baseline participant information (participant demographics, smoking history, beliefs about smoking, and readiness to quit), randomized arm, and the number of minutes spent on the first core content page viewed by the participant. Additionally, we used, as predictors, information about the type of webpage viewed, such as the content addressed in the webpage (getting ready to quit, quitting, and staying quit) and the type of page viewed (eg, introduction page, testimonial).

### Effect of Content Tone on Engagement

We calculated the total number of intervention visits, individual page views, and total number of page views that resulted in a time-out. We summarized the distribution of the time in minutes that participants spent viewing intervention content excluding all timed-out page views. After imputing missing page view times, total time spent online was calculated for each participant by adding up the number of minutes spent on an intervention webpage. In order to evaluate the impact of assigning an arbitrary value for time spent viewing pages that resulted in a time-out, we varied the number of minutes assigned to page views that timed out from near zero to 30 minutes.

We then compared the contrasting factor levels of message tone on the total time spent viewing intervention content using a zero-inflated Poisson (ZIP) model [40,41]. We used a ZIP model because the distribution of total time spent online had a larger proportion of zeros than expected from a Poisson distribution; study subjects who were never exposed to the intervention content all spent exactly zero total minutes online, causing a notable point mass in the distribution at zero. We included in the logistic portion of the ZIP, which models the “excess” zeros in the population, an intercept term. In the Poisson part of the ZIP model, we included the randomized factor level and the baseline readiness to quit measure that was used to stratify randomization. We report the estimates from the Poisson part of the ZIP model. Generally, estimates obtained from Poisson models are interpreted as incidence rate ratios, but when all subjects share a common period of exposure, as in the Q^2^ trial, estimates can be interpreted as the ratio of mean event counts comparing the two contrasting factor levels. Thus, we report the ratio of the mean number of minutes spent online for individuals who received the content in a motivational tone to those who received the prescriptive message tone. We used Stata Version 12 for all analyses, including imputing missing page view times [30,42].

## Results

**Figure 1:**
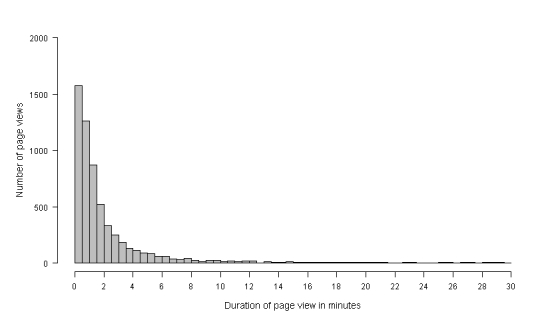
Distribution of minutes spent viewing an intervention page, excluding page views that resulted in an automatic time-out.

The Q^2^ trial enrolled 1865 current smokers; 1175 (63.00%, 1175/1865) participants accessed the online intervention at least once. The intervention content was viewed on a total of 1691 separate visits, resulting in 6592 unique page views. A total of 683 (10.36%, 683/6592) of these page views automatically timed out after 30 minutes of inactivity, and 550 (46.81%, 550/1175) participants had at least one page view that resulted in a time-out. Figure 1 shows the distribution of the time spent on page views that did not result in a time-out; the median observed time spent on an intervention page was 1.07 minutes (interquartile range 0.47-2.27). This suggests that assigning 30 minutes to all page views that resulted in a time-out would overestimate the time participants spent viewing online intervention content.

**Figure 2:**
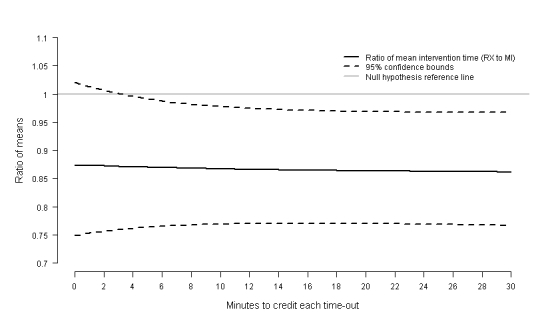
Sensitivity of model results to assigning an arbitrary time spent online to page views that resulted in a time-out (estimate from the zero-inflated Poisson model for the ratio of the mean time spent online comparing individuals who received content in a prescriptive [RX] tone versus a motivational tone [MI]).

Figure 2 presents the estimated ratios of mean time spent online for those who received content in a prescriptive tone compared to those who received content in a motivational tone when the value assigned to the time spent viewing webpages that resulted in a time-out is varied from near zero to 30 minutes. While the ratio of means estimate was stable around 0.87, the width of the 95% confidence intervals (CI) around the estimate vary as the time assigned to time-outs changes. Assigning a value close to zero (0.00001 minutes) for time-outs resulted in an estimate of 0.87 with a 95% CI 0.75-1.02 that includes one (ie, fail to reject the null hypothesis that there are no differences in participant engagement between the two factor levels at a 0.05 significance level). Alternatively, assigning a value of 30 minutes to page views that automatically timed out resulted in an estimate of 0.86 with a 95% CI 0.77-0.97 that excludes one, leading to the conclusion that participants assigned to the prescriptive tone viewed content for significantly fewer minutes than those assigned to the motivational tone.

Averaged across the five completed datasets (ie, time-outs replaced with imputed page view times), the average total time spent viewing intervention content was 12.3 minutes. The total number of minutes spent viewing the intervention ranged from less than 1 minute to greater than 180 minutes, with a median of 7.0 minutes. Comparing the mean cumulative number of minutes spent viewing intervention content among those who viewed content in a prescriptive tone versus a motivational tone resulted in a ratio of means of 0.87 (95% CI 0.75-1.01; *P*=.06). Thus, participants who had content presented in a prescriptive tone spent 13% less time viewing online intervention content, although this difference was not statistically significant at the .05 level.

## Discussion

### Principal Findings

The number of available Internet-based behavioral and educational intervention programs has exploded over the past decade. As researchers seek to understand how to optimize the design of these programs to be most effective, it is imperative that researchers examine to what extent participants are exposed to and engage with the programs and to what extent this interaction influences intervention outcomes. Even with the advent of more sophisticated means for tracking program interactivity, there will continue to be periods of time which, either by design or happenstance, involve no direct human-computer interactions resulting in extended periods of “inactivity”. As our case example illustrates, how these data are handled analytically can significantly alter the conclusions drawn about how much time participants actually spent viewing the content. In turn, this could affect analyses designed to explore whether or not program exposure mediated the observed treatment effects.

### Conclusions

We propose a standard methodology whereby researchers utilize the MI processes outlined in this paper for managing extended periods of inactivity or time-out data. The decision to use this methodology should be made a priori, as one cannot know ahead of time how much of an impact assigning an arbitrary value to time-outs will have on study conclusions. Researchers are encouraged to employ multiple imputations when examining exposure to online intervention content in the future.

## Multimedia Appendix 1

Q^2^ screenshot.

## Abbreviations

MAR: missing at random
MCAR: missing completely at random
MI: multiple imputations
MICE: multiple imputations by chained equations
NMAR: not missing at random
ZIP: zero-inflated Poisson model
