# Supplementary figures and images for: Using Multiple Imputations to Accommodate Time-Outs in Online Interventions
Source: J Med Internet Res. 2013 Nov 21;15(11):e252. doi: 10.2196/jmir.2781 (PMC3841344; doi:10.2196/jmir.2781)

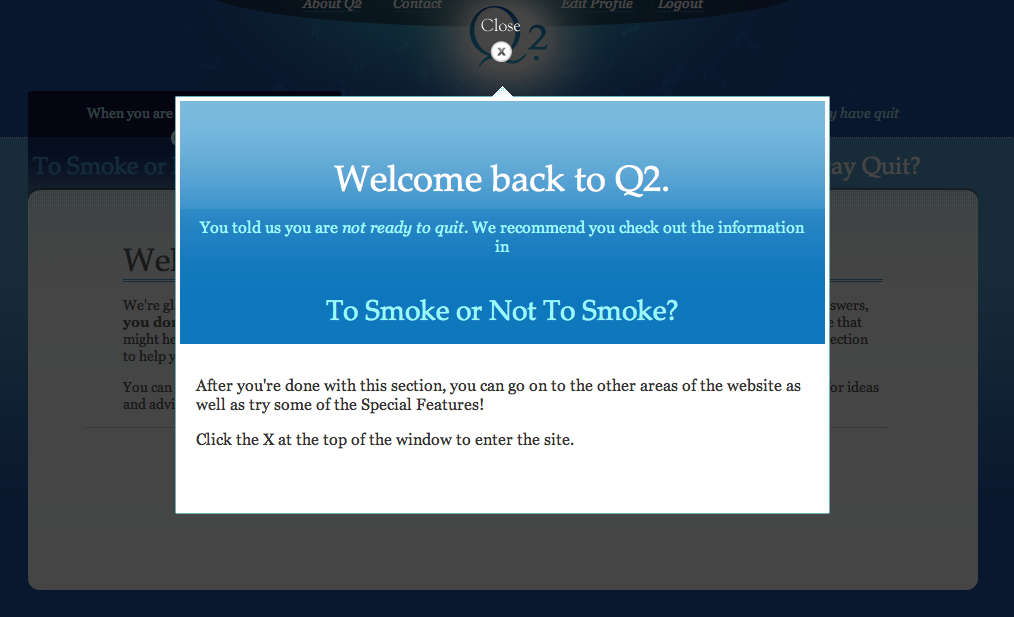

Supplement: Supplementary file 1 [file jmir_v15i11e252_app1.png]
